# Ethical decision-making under stress: the role of the nonreactive facet of mindfulness

**DOI:** 10.3389/fpsyg.2026.1879880

**Published:** 2026-08-12

**Authors:** Sheila K. Hanson, David Hollingworth, Sean R. Valentine

**Affiliations:** Middleton School of Entrepreneurship & Management, Nistler College of Business & Public Administration, University of North Dakota, Grand Forks, ND, United States

**Keywords:** business ethics, ethical decision-making, mindfulness, nonreactive mindfulness, perceived stress

## Abstract

Business students commonly experience high levels of stress, often facing a variety of ethical challenges throughout their education experiences and well into their professional lives. Complicating matters, past research shows that such stress may compromise the processes associated with individual ethical decision-making (EDM). Some research indicates that individual differences, namely mindfulness, can benefit EDM. However, few if any studies have considered how mindfulness may mitigate adverse effects of stress on EDM. Many models of the EDM process have assumed a primary role for cognitive reasoning, while many models of moral decision-making explicitly consider intuitive responses as a dominant factor. Drawing on dual-process theory, we propose that perceived stress undermines the EDM process by shifting processing away from controlled, deliberative cognition toward more automatic, intuitive responses. Critically, the nonreactive facet of mindfulness serves as a mediating mechanism that enables individuals to pause these automatic reactions and engage in more reflective evaluation. Mindfulness is a multi-faceted construct, and its nonreactive facet is of particular interest because it may delay automatic intuitive responses, thereby facilitating cognitive evaluations, prior to response. We empirically evaluated competing hypotheses that the nonreactive facet of mindfulness would either partially or fully mediate the effect of stress on the EDM process in a sample of 261 Midwestern USA university business students. Structural equation modeling and mediation analysis supported a fully mediated model, suggesting that nonreactive mindfulness mediates the relationship between stress and the EDM process among business management students. Theoretical and practical implications are discussed, as are the study’s limitations and suggestions for future research.

## Introduction

“Scandals in business never seem to end. Even when one scandal seems to finally end, another company outdoes the prior disgraced company and dominates the public dialog on corporate ethics” (DeTienne et al., 2021, p. 429). The workplace continues to be fraught by unethical activity despite efforts to increase the presence of ethics codes and training programs. In national workforce survey, 41% of responding workers reported observing ethical misconduct on the job, with 60 percent of the observed misconduct involving those with “managerial authority from supervisory level up to top management” and top management constituted nearly one quarter (24%) of the observed misconduct (Ethics Resource Center [ERC], 2014, p. 11). More recently, a Gallup poll asked respondents to rate the honesty and ethical standards of business executives and found that 42% of respondents rated business executives as low or very low (Brenan and Jones, 2024).

Ethical failures don’t simply occur within work contexts—unethical attitudes and behaviors are also experienced in business education environments (Klein et al., 2007; Parks-Leduc et al., 2022; Siala et al., 2025). Haski-Leventhal et al. (2022, p. 17), discussing comments from Kidwell and Scherer (2001), noted that “…for students, the line between right and wrong was increasingly blurred and that students expected managers to behave unethically.” Students will inevitably encounter ethical dilemmas as their careers unfold. Preparation to respond to these dilemmas shapes their ethical development (Martinov-Bennie and Mladenovic, 2015; Mladenovic et al., 2019). Yet, there is limited understanding about the EDM process in individuals preparing to or entering the workplace (Weber, 2024).

EDM is consequential because its effects extend beyond the individual decision-maker. Unethical decisions may result in personal, professional, legal, and reputational consequences for actors, while simultaneously imposing substantial costs on organizations and society (Treviño et al., 2014). Organizational misconduct can damage employee trust, undermine organizational effectiveness, expose firms to financial and legal liabilities, and erode public confidence in business institutions (Kaptein, 2011; Treviño et al., 2014). At the societal level, highly publicized ethical failures contribute to broader economic and social harms and can diminish trust in business leaders and organizations (DeTienne et al., 2021). Since many business students will eventually assume roles in organizations where their decisions affect employees, customers, investors, and communities, management educators have an important responsibility to prepare students to recognize and effectively navigate ethical challenges (Haski-Leventhal et al., 2022; Martinov-Bennie and Mladenovic, 2015). Moreover, ethically consequential decisions are frequently made under conditions of stress, uncertainty, and competing demands rather than ideal circumstances (Selart and Johansen, 2011). Consequently, identifying factors that help individuals maintain the EDM process under stress has important implications for management education, organizational practice, and broader societal well-being.

Concerns about managerial ethics and EDM are sufficiently widespread that business and management education accreditation bodies are seeking to institutionalize “…a deeper understanding of corporate and social responsibility and business ethics into the business management curricula” (Siala et al., 2025, p. 571). Haski-Leventhal et al. (2022) argue that responsible management education should provide students with more than just managerial knowledge to facilitate the development of responsible managers. In addition to learning about ethical frameworks, students (and working professionals) may benefit from understanding how various factors are likely to affect their own EDM.

Commonly accepted models of the EDM process in business generally present it as a series of sequential stages in the following temporal order—recognition of an ethical issue leading to a judgment about the issue, followed by the development of intentions to act, culminating in behavioral outcomes (e.g., Dubinsky and Loken, 1989; Ferrell and Gresham, 1985; Hunt and Vitell, 1986; Jones, 1991; Rest, 1986; Treviño, 1986). This conceptualization of the EDM process follows a rationalist perspective that emphasizes structured reasoning. In contrast, other theories involving moral decision-making focus on the role of intuition and emotion as primary forces in the process (e.g., Haidt, 2001; Sonenshein, 2007). From this perspective, moral decision-making is more unconscious and automatic and may not involve much intentionality (Haidt, 2001; Ming et al., 2025). Dual-system theories integrate cognitive and intuitive/emotional views and propose that both reasoning and intuitive, automatic factors can result in ethical or unethical behavior (Dedeke, 2015; Haidt, 2001; Reynolds, 2006; Schwartz, 2016). Dual-system theory suggests that individuals may enact both reasoning and intuitive systems in the EDM process (Haidt, 2001; Ming et al., 2025).

Recent studies suggest that mindfulness may operate to affect cognitive and/or intuitive/emotional processes involved in the EDM process (Ming et al., 2025; Ruedy and Schweitzer, 2010; Small and Lew, 2019). Baer et al. (2006, p. 27) define mindfulness as “…bringing one’s complete attention to the experiences occurring in the present moment, in a nonjudgmental or accepting way.” Mindfulness positively influences memory, concentration, and a plethora of other work domains (Good et al., 2016) and may influence the development and practice of EDM. Mindfulness is a self-regulatory approach to experiencing life with a present-moment orientation of curiosity, openness, and acceptance (Bishop et al., 2004; Kabat-Zinn, 1990). Mindfulness entails cognitive and emotional approaches, whereby individuals are attentive to events and experiences yet remain flexible in how they react to and interpret them (Bishop et al., 2004). Over decades of accumulating evidence, mindfulness is known for its efficacy in managing life and work stressors as well as both acute and chronic stressful situations in various life domains including the workplace (Jamieson and Tuckey, 2017; Lomas et al., 2019; Vonderlin et al., 2020). Broadly, mindfulness is a self-regulatory process with both meta-cognitive and meta-affective qualities (Vago and Silbersweig, 2012). Mindfulness is recognized as both a trait and state, particularly malleable through brief and longer-term training programs and is associated with positive outcomes in the domains of cognition and emotion regulation (Creswell and Lindsay, 2014; Good et al., 2016). Although mindfulness overlaps conceptually with emotional intelligence and emotion regulation, the nonreactive facet of mindfulness, the focus of the current study, plays a distinct role in the regulation process. Emotion regulation refers broadly to strategies for managing or modifying emotional experiences, whereas nonreactive mindfulness specifically involves the ability to observe thoughts and emotions without automatically responding to them. This capacity creates space for more deliberate and adaptive responses (Baer et al., 2006; Good et al., 2016; Gross and John, 2003). Importantly, mindfulness is amenable to change as it may be learned and cultivated through practice.

Results of a comprehensive review (Good et al., 2016) and meta-analysis (Mesmer-Magnus et al., 2017) involving individuals in the workplace suggest that mindfulness is associated with a myriad of positive outcomes for paying attention, managing emotions, and work performance. There is accumulating evidence that mindfulness precipitates improved problem solving (Ostafin and Kassman, 2012) and decision-making (Good et al., 2016). However, the literature on mindfulness and the EDM process is sparse.

Two studies using the more common cognitive approach to EDM, considered mindfulness as an individual factor. Each of these considered the influence of mindfulness on a limited portion of the EDM process (Ruedy and Schweitzer, 2010; Small and Lew, 2019). Another study investigating mindfulness and EDM used a dual-process model of both cognitive and intuitive processes (Ming et al., 2025). Some research has identified specific ethical cognition processes associated with mindfulness, including forecasting consequences and perceptions of moral intensity (Stenmark and van Ittersum, 2025). All of these studies conceptualized mindfulness as a global construct, so little is known about whether specific facets of mindfulness, such as the nonreactive facet, play a unique role in EDM under conditions of stress. None of these studies considered how specific facets of mindfulness might uniquely covary with EDM, even though these components can prompt differential effects (e.g., Baer et al., 2006; Haenen et al., 2016). Although mindfulness has typically been examined as a global construct in prior research, its multifaceted nature suggests that different components may operate through distinct mechanisms. Among these, the nonreactive facet is especially relevant in high-stress ethical contexts because it directly governs the inhibition of automatic responses—an ability central to dual-process accounts of decision-making. Accordingly, we focus specifically on nonreactive mindfulness as the key mechanism through which stress may influence EDM.

Stress is pervasive for college students (American College Health Association [ACHA], 2020; Kuhnell et al., 2020) and young professionals (e.g., American Psychological Association [APA], 2024; Nova, 2023). Selart and Johansen’s (2011) study found evidence to support the premise that stressful events (in general) influence leaders’ recognition of moral issues, although their results did vary situationally. However, they did find evidence of a negative effect (for stress) on the establishment of moral intent (Selart and Johansen, 2011). Their results also suggest that stressful events may differentially affect various aspects of the EDM process. Another study found that decision-makers under chronic stress tended to use less cognitively challenging decision approaches or delayed EDM (Zhang et al., 2018). Lawrence and Kacmar (2017, p. 39) found that emotional exhaustion resulting from stress “impairs an employee’s ability to activate self-regulating processes to avoid engaging in unethical behavior.” A study of “star” employees found that role stress increased moral disengagement and unethical behaviors (Xue et al., 2024). As a group, the studies indicated adverse effects of stressful events and/or chronic stress on moral engagement, recognition of moral issues, ethical intent and ethical behavior. Using various theoretical bases, they reasoned that stress (and stressful events) compromise cognitive and emotional resources and capacity, while reducing information-gathering ability by impairing working memory and reducing concentration. Enhanced levels of stress may lead to a greater reliance on intuitive or emotion-based decision-making, which also may be compromised.

Research on externally-imposed performance pressure (e.g., Stenmark and Kreitler, 2019) suggests similar effects on EDM with demanding performance expectations, time constraints, and evaluative environments can create urgency, narrow attentional focus, and heighten emotional responses, thereby impairing deliberative processing and increasing reliance on more immediate, automatic reactions. Stenmark and Kreitler’s (2019) study focused on situationally-induced pressure rather than perceived stress. In contrast, the current study focuses on perceived stress as the individual-level appraisal of environmental demands, irrespective of their source. This approach allows us to capture the extent to which individuals perceive that situational requirements exceed their capacity to cope, whether those demands originate from externally imposed pressure, internal expectations, or broader contextual factors. Accordingly, perceived stress provides a theoretically appropriate lens through which to examine how such conditions influence the EDM process.

We build upon previous EDM research with a novel model which incorporates both perceived stress and nonreactive mindfulness, informed by the dual-process model approach to EDM. Our conceptualization provides for individually perceived stress to influence the EDM process directly and allows a stronger role for intuitive processes and a lesser role for intuitive processes. As the facets of mindfulness can differentially mediate various effects (e.g., Haenen et al., 2016), we allow for a particularly relevant facet of mindfulness, the nonreactive facet, to operate as a mediator through which relatively automatic (intuition-based) EDM responses are delayed sufficiently to allow for cognitive processing which considers both intuitive and non-intuitive factors as the EDM process unfolds. As such, it suggests a dual path for the influence of ongoing perceived stress while acknowledging the potentially positive effects of nonreactive mindfulness, in the presence of stress.

The current study aims to expand the existing literature by investigating the role of mindfulness in relation to the EDM process of university students in management courses, many of whom are also working adults in the early stages of their careers. Regardless of the career path they pursue, they will inevitably be faced with ethical situations in the workplace. There is limited workplace literature (Ming et al., 2025; Ruedy and Schweitzer, 2010; Small and Lew, 2019) on mindfulness, and none of the existing literature appears to consider how the nonreactive facet of mindfulness (i.e., nonreactive mindfulness) might operate as an antecedent of EDM, particularly in the context of business education.

## Literature and hypotheses

### Perceived stress and nonreactive mindfulness

The American College Health Association (American College Health Association [ACHA], 2020) indicates that nearly 75% of US college students experienced moderate (49.8%) to high (25.1%) levels of stress. Academic workload, financial strain, and social pressures are common stressors. The COVID-19 pandemic exacerbated stress with uncertainty, remote work and learning challenges, and social isolation (Son et al., 2020; Wang et al., 2020). Wang et al. (2020) found that average perceived stress scores among university students during the pandemic was high. Recent studies also indicate that high stress levels among young professionals are similarly elevated (American Psychological Association [APA], 2024; Nova, 2023). In the current study, perceived stress is defined as “the perceived degree to which environmental demands exceed abilities to cope” (Cohen and Williamson, 1988, p. 37). Stressors are conditions that create strain on the person resulting in psychological, physiological, and behavioral outcomes (Bliese et al., 2017). In the present study, the effects of stress on mindfulness are of particular interest.

Mindfulness is multi-faceted and those facets are differentially correlated with other constructs (Baer et al., 2006). Baer et al. (2006, p. 38) noted that “measurement…at the facet level is useful for understanding their relations with other variables.” Being nonreactive to one’s inner experience is a particularly relevant facet of mindfulness, when considering dual process theories of EDM. This involves recognition of one’s intuitive/emotional response (to a potentially morally challenging situation) while simultaneously withholding automatic response; thereby allowing cognitive consideration as well as intuition and emotions. The nonreactive facet refers to remaining calm and objective despite difficult thoughts and feelings, remaining present in the moment, and emotionally regulated (Bishop et al., 2004). Thus, nonreactive mindfulness is a self-regulatory process consisting of managing reactions in response to environmental stimuli or internal mental events (Baer et al., 2006).

Empirical findings link mindfulness to psychological and physiological processes via the stress response, whereby mindfulness is related to neurobiological mechanisms involved in stress regulation (Creswell and Lindsay, 2014), including dampened stress reactions in response to cognitive and social threats and faster recovery to baseline levels (Brown et al., 2012). However, in the absence of a mindfulness intervention which may moderate stress response via manipulation of state mindfulness, the presence of stress may adversely impact trait mindfulness. In this study, our focus is on the individual’s perceived stress (from whatever sources) and non-manipulated (trait) mindfulness, specifically the nonreactive aspect of mindfulness.

Numerous theories suggest and existing studies confirm that higher levels of stress adversely affect cognition via reduced cognitive resources and capacity, which, in turn, would be expected to impair the ability to withhold reaction. Selart and Johansen (2011) reasoned that stress would result in impaired working memory and reduced concentration, while Lawrence and Kacmar (2017), p. 39) found that stress “impairs an employee’s ability to activate self-regulating processes to avoid engaging in unethical behavior.” Zhang et al. (2018) found that decision-makers under chronic stress tended to use less cognitive challenging decision approaches or delayed EDM as coping mechanisms. Finally, Xue et al. (2024) found increased moral disengagement in response to higher levels of stress. These are only a few of many studies which provide insight into how stress may affect the nonreactive aspect of (trait) mindfulness. This leads us to the following hypothesis:

H1: Perceived stress is negatively related to nonreactive mindfulness.

### Nonreactive mindfulness and the EDM process

Schwartz (2016) proposed an integrated EDM model, combining cognitive, intuitive, and emotional components. The cognitive perspective is based in Kohlberg’s (1976) three stages of moral development: (1) pre-conventional, wherein the decision-maker is primarily motivated to avoid punishment and fulfill self-interest; (2) conventional, wherein the decision-maker considers referent others and existing law; and (3) post conventional, where the decision-maker utilizes social contracts and universal ethical principles to inform decision-making. Rest (1986) built on this perspective specifying four distinct stages of the EDM process, beginning with awareness of a moral or ethical issue, reaching a judgment concerning the issue, developing an intended response, and culminating in action or behavior.

However, as the dual-process models suggest, individuals may engage in EDM “in a more unconscious manner than the more conscious, controlled steps of moral reasoning presented in Kohlberg’s more rationalist model of moral judgment “ (Elm and Radin, 2012, p. 316). The intuitive perspective (e.g., Ruedy et al., 2013; Saltzstein and Kasachkoff, 2004) often invokes the role of emotion; however, it also allows for limited cognitive response via intuition (e.g., Haidt, 2001) or cognitive script processing (e.g., Hollingworth, 2014; Treviño and Nelson, 2011, p. 102). This view acknowledges that EDM is often complex and frequently occurs in the moment, while relying on prior experiences. A common thread of the intuitive approach is that rational decision-making is pre-empted by more immediate processes.

Two studies suggest that mindfulness promotes EDM by enhancing cognitive interpretation of the moral aspects of decision-making processes (Ruedy and Schweitzer, 2010; Small and Lew, 2019). Taken together, these studies suggest that mindfulness helps individuals remain present and reflective, thereby reducing impulsive responses and fostering a stronger commitment to ethical principles. Both studies took primarily a cognitive approach. However, EDM may involve both cognitive and affective elements. Schwartz’s (2016) model allows for situational and individual factors to operate on and in-between all stages of EDM. One such individual factor that we consider is mindfulness, specifically nonreactive mindfulness which may influence both the rationalist (cognitive) and non-rationalist (intuitive and affective) elements.

Nonreactive mindfulness is a self-regulatory process consisting of managing reactions in response to environmental stimuli or internal mental events (Baer et al., 2006). It plays an important role in decision-making, particularly in complex or emotionally charged situations (Gross and John, 2003). Research shows that individuals who are better able to regulate their emotions are less influenced by emotional biases and more likely to make effective decisions (Gross and John, 2003). For example, individuals with higher levels of nonreactive mindfulness may be better able to resist the influence of emotionally charged messages or persuasive arguments that are designed to elicit an emotional response (Gross et al., 2006).

Nonreactive mindfulness harnesses emotion regulation and present-moment cognition and may also aid in mitigating the negative effects of stress on decision-making. Task or situation-related emotion may sometimes benefit decision-making, but unrelated emotions can disrupt decision-making (Preston et al., 2007). Individuals who are anticipating stress may move toward effective decision-making more slowly than individuals experiencing less stress (Preston et al., 2007), as well as individuals currently experiencing stress (Zhang et al., 2018). Individuals who are better able to regulate their emotions may be less susceptible to the negative effects of stress on decision-making (Gross et al., 2006). Thus, nonreactive mindfulness plays an important role in decision-making by helping individuals resist cognitive and emotional distractions and biases facilitating more effective decisions, despite complex or emotional situations.

Importantly, nonreactive mindfulness may enable individuals to approach ethical situations with a calmer mindset, resilient to the demands of both chronic and situational stressors. In the face of challenging ethical situations and scenarios, reactive emotions such as anger and fear might impair the EDM process and lead to impulsive decisions. Nonreactive mindfulness, as a self-regulatory process, involves regulating emotional responses with the intention of maintaining cognitive clarity and objectivity, allowing individuals to step back from immediate thoughts and emotional reactions. Although that reflective “pause” may be brief, it is powerful (Kabat-Zinn, 2001). Nonreactive mindfulness allows individuals to more carefully consider the various dimensions of a decision, possible courses of action and make decisions guided by ethical principles rather than expressing reactive thoughts and emotions. Further, nonreactive mindfulness is also beneficial in more intuitive and emotional processes (Kabat-Zinn, 2001). Overall, nonreactive mindfulness is expected to positively influence EDM by fostering a calmer mindset, helping individuals manage emotions, and facilitating enhanced consideration of ethical issues.

H2: Nonreactive mindfulness is positively related to the EDM process.

### Perceived stress and the EDM process

Stress influences individuals’ behavior, cognition, emotion, and decision-making (Starcke and Brand, 2012). In stressful environments, common to young adults, including in academic settings and the workplace, many decisions are made under chronic or acute stress. Heightened stress can influence cognitive functions such as attention, executive functioning, and associated processes such as cognitive flexibility, which influence the higher-order cognition needed for EDM. According to Reynolds (2006), neurocognitive research suggests that EDM involves a complex process of reflexive matching of current situations with prior prototypes and higher order conscious reasoning, whereby individuals process not only their immediate perceptions of the environment but also their complex thoughts about those perceptions. However, as noted by Sandi (2013), stress can negatively affect the prefrontal cortex, which is essential for higher-order cognitive processes associated with reasoning involved in the EDM process. In young adults, the prefrontal cortex is not fully developed until the mid-20s or later (Johnson et al., 2009), which could lead to difficulties in processing complex ethical dilemmas and consequences. Further, stress can amplify emotional responses and biases, leading to more impulsive and less reasoned decision-making. Stress could be associated with decisions driven by immediate emotional reactions rather than thoughtful consideration of ethical standards (Starcke and Brand, 2012). Under stress, individuals may be influenced by their emotions rather than carefully considering ethical decisions. Thus, emotional reactivity may lead to impulsive and less ethical choices.

Overall, perceived stress may negatively impact the EDM process in numerous ways. Kouchaki and Desai (2015) found that stress and fatigue are associated with decreased ethical behavior, as individuals under stress are more likely to engage in unethical actions to cope. Chronic stress is associated with an increased tendency toward deontological moral choices, prioritizing adherence to moral rules and duties over considering the consequences of actions (Zhang et al., 2018). A rigid approach to moral reasoning can hinder the ability to weigh situational factors and make nuanced ethical judgments. In addition, stress may influence EDM when emotion is influential. Kligyte et al. (2013) found that anger reduced EDM, while emotion regulation attenuated those effects. Simlarly, Thiel et al. (2011) found that appraisal involving anger negatively influenced the use of metacognitive strategies and ethical decisions. Taken together, these findings suggest that stress may affect EDM by both depleting access to cognitive resources and increasing emotionally driven, less reflective decision-making.

Perceived stress creates a sense of urgency and pressure, which may lead to rushed decision-making without adequate ethical consideration. The pressure to make quick decisions can overshadow the importance of deliberation. Understanding how stress influences EDM is important toward improving EDM and ultimately, ethical decisions (Selart and Johansen, 2011). We expect the following relationship between perceived stress and EDM:

H3a: Perceived stress is negatively related to the EDM process.

### Nonreactive mindfulness as a potential mediator of perceived stress and the EDM process

To the best of our knowledge, prior research has not considered relationships between nonreactive mindfulness, perceived stress and the EDM process. Stress constitutes the relationship between a set of external stressors and the individual’s ability to cope with these stressors resulting in psychological and physiological effects. Mindfulness allows for a mental space between a stimulus (e.g., an ethical situation) and a response, meaning that rather than “reacting” to a stimulus, individuals have more space to “respond” (Good et al., 2016). Thus, nonreactive mindfulness is a key mechanism in this process. Considering the nuances and complexity of ethical situations, approaching them with nonreactive mindfulness can improve cognitive resources for reasoning as well as reduce automatic intuitive responses to benefit the EDM process. In the current study, we consider nonreactive mindfulness as a potential mechanism to buffer the relationship between perceived stress and the EDM process leading to the following hypothesis:

H3b: The negative relationship between perceived stress and the EDM process is mediated by nonreactive mindfulness.

## Materials and Methods

### Participants

Participants were students enrolled in undergraduate and graduate business management classes, a majority of whom were working in some capacity for an employer. A survey was conducted via Qualtrics and incentivized with extra-credit points. Due to survey length, multiple response/attention-checks were used, and some instances of disengagement were detected. A total of 402 students initiated the survey generating 352 provided usable responses. Due to attention-check failures, the finalized data set contained 261 respondents.

### Demographics

Respondents were students taking business management-related courses at a midwestern university located in the United States. Ages ranged from 18–50 years old, with an average age of 22.5. Reporting valid percentages, 64.8% of individuals indicated that their biological gender was male, and 35.2% of them reported that their biological gender was female. Respondents were primarily single (90.4%), 8.5% were married, and 0.8% were separated or divorced. The highest level of education completed was a graduate degree (1.1%), while others indicated that they had completed some graduate work (10.0%), a Bachelor’s degree (8.8%), some college (75.1%), or a high school diploma (5.0%). Most participants identified as White (92.3%) followed by Black (2.7%), Asian (2.7%), and Hispanic (0.8%) with 1.5% endorsing “Other.” Regarding employment status, 95.4% of the sample responded while 4.6% did not. Of the respondents, one-fifth indicated that they were full-time employees, the majority (62.0%) were part-time employees, and 18% were temporary employees. About one-sixth of survey respondents (17.3%) reported that they were currently a manager or supervisor in their organizations. If they were presently working, respondents indicated their primary work classifications, which included Customer Service (17.7%), Sales/Marketing (15.9%), Accounting (6.2%) and General Management (5.8%), and “Other” (36.7%). Over two-thirds (69.5%) of individuals reported that their company had shared with them an ethics code that governs work conduct in the organization.

## Measures

### Perceived stress

Rather than providing study participants with potentially stressful situations or events and attempting to estimate the degree of stress experienced, this study measured the level of stress participants were experiencing, irrespective of source(s). Individuals’ perceptions of stress were measured using the 10-item Perceived Stress Scale (Cohen et al., 1983; Cohen and Williamson, 1988). Sample questions include “In the last month, how often have you felt nervous and “stressed”?” and “In the last month, how often have your felt difficulties were piling up so high that you could not overcome them?” Individuals provided responses to these questions with a five-point scale (Never = 0, Almost Never = 1, Sometimes = 2, Fairly Often = 3, Very Often = 4). After reverse scoring four items, higher scores indicated elevated levels of stress. Factor analysis using principal component extraction and pairwise deletion with the model constrained to a single factor yielded an initial eigenvalue of 4.56, 45.60% of explained variance, and nine items with loadings greater than 0.610. One item had a loading of 0.556 (“In the last month, how often have you been able to control irritations in your life?”) and was deleted from the model. A similar second factor analysis yielded an initial eigenvalue of 4.30, 47.82% of explained variance, and nine items with loadings greater than 0.580. The items also demonstrated adequate internal consistency reliability (α = 0.86).

### Nonreactive mindfulness

Nonreactive mindfulness was measured using the nonreactive items from the Five-Facet Mindfulness Questionnaire (Baer et al., 2006). Sample items are “When I have distressing thoughts or images, I “step back” and am aware of the though or image without getting taken over by it” and “When I have distressing thoughts or images, I just notice them and let them go.” Respondents used a five-point scale (Never or very rarely true = 1, Rarely true = 2, Sometimes true = 3, Often true = 4, Very often or always true = 5) to rate these items. A principal component-based factor analysis using pairwise deletion and constrained to one factor yielded an initial eigenvalue of 2.82, 40.29% of explained variance, and five items had loadings greater than 0.600. Two items with a loading of 0.580 (“I perceive my feelings and emotions without having to react to them”) and 0.573 (“In difficult situations, I can pause without immediately reacting”) were deleted from the model. A second factor analysis with the same criteria had an initial eigenvalue of 2.32, 46.44% of explained variance, and five items with loadings greater than 0.630 and sound reliability (α = 0.71).

### EDM process

As previously noted, dual process theories allow for stress and nonreactive mindfulness to affect various stages of the EDM process. Our primary interest was in how these variables affect the EDM process overall rather than any specific stage of the EDM process. Therefore, the EDM process was measured holistically by combining measures of the individual’s recognition of an ethical issue, judgments about the ethical issue, and behavioral intentions associated with the ethical issue.

Organization-based EDM research often utilizes scenarios to explore various ethical issues (e.g., Alexander and Becker, 1978; Barnett, 2001; Reidenbach and Robin, 1990; Singhapakdi et al., 1996). Since a single vignette may elicit responses specific to a particular issue, we followed prior research (e.g., Barnett, 2001; Barnett and Valentine, 2004; Hollingworth and Valentine, 2015; Reidenbach and Robin, 1990; Valentine and Hollingworth, 2012) by including multiple ethics-related scenarios. Assessing reactions across diverse issues enables a more comprehensive evaluation of the EDM process and improves the generalizability of the findings. Participants were presented three separate scenarios highlighting morally questionable business situations and actions, each of which was followed by the same measures prompting individual assessments linked to different components of the EDM process. An adapted version of a previously published scenario (“Jane”; Valentine and Hollingworth, 2012) and two previously unpublished scenarios (“Jerry” and “Mary”) were used:

Situation: Jane manages a call center that is experiencing poor operational efficiency. Continued poor performance might cause Jane to lose her annual bonus. Jane’s boss suggests either a) reduce staff by two workers - which would be a “quick fix,” or b) provide additional training for all workers, which will take more time and effort to implement. All call center employees are hard-working, have received good performance reviews, and really need their jobs.
Action: Two days later, Jane calls two workers into her office to give them the bad news - they no longer work at the call center.
Situation: Jerry is an entrepreneur who owns and operates a sporting goods business. His firm uses one supplier for all screen-printed T-shirts. The most recent shipment of T-shirts had a minor quality-related problem that could be fixed quickly and at very little expense. Jerry had written the purchase contract with intentionally vague (unclear) details about how quality-related problems would be resolved.
Action: When Jerry called the supplier to discuss the recent shipment, he said, “The most recent shipment of T-shirts completely failed our quality inspection! We can fix them, but we will require a 60% discount on this shipment to pay us for salvaging these T-shirts. If you will not agree to that, you should know that I will probably have to switch our T-shirt purchases from you to our other T-shirt supplier.”
Situation: Mary is trying to decide whether she should place an order with supplier “A” or supplier “B.” Supplier “A” has a price that is a little lower, and their quality is slightly better than that of Supplier “B.” However, she knows her boss has always preferred Supplier “B,” although she is not sure why. Mary has developed a personal friendship with the salesperson at Supplier “B” and they get along quite well.
Action: Previously, Mary had written the request for bids/price quotes that favored supplier “B.” Today, Mary decided to place the order with Supplier “B.”

Recognition of an ethical issue was measured with the question “Do you believe that this scenario involves an ethical issue?” that was rated with a seven-point scale with opposing anchors (Completely Disagree this scenario involves an ethical issue = 1 to Completely Agree this scenario involves an ethical issue = 7) (Barnett and Valentine, 2004). Similar to procedures used in past education-based business ethics research (Barnett et al., 1994; Silver and Valentine, 2000), the ethical issue recognition scores for each of the scenarios were averaged for a more comprehensive measure, with higher overall values indicating stronger ethical issue recognition. Ethical judgment was assessed using four items measuring “moral equity” (Reidenbach and Robin, 1990), which were preceded by a prompt to evaluate the actions described in the situations with four sets of opposing anchors (Fair/Just/Morally right/Acceptable to my family = 1 to Unfair/Unjust/Not morally right/Unacceptable to my family = 7). Items scores were averaged to show stronger ethical judgments across the three ethics scenarios (Jane scenario α = 0.93, Jerry scenario α = 0.95, Mary scenario α = 0.92), and composite scores were averaged to obtain an overall value. Ethical intention was measured by asking individuals how likely they would engage in the actions described in the situations, followed by four seven-point scales with opposing anchors (Likely/Probable/Possible/Definitely would = 1 to Unlikely/Improbable/Impossible/Definitely would not = 7) (Barnett et al., 1996; Barnett and Valentine, 2004; Bass et al., 1999). Items scores were averaged to indicate a stronger ethical intention across the vignettes (Jane scenario α = 0.96, Jerry scenario α = 0.97, Mary scenario α = 0.96), and composite scores were averaged for an overall value. A principal component factor analysis of the overall values using pairwise deletion produced an initial eigenvalue of 2.14, 71.48% of explained variance, and loadings greater than 0.710.

#### Social desirability

Social desirability can adversely impact business ethics studies given the tendency of respondents to provide favorable opinions (Randall and Fernandes, 1991). Consequently, a 10-item measure of social desirability (Crowne and Marlowe, 1960; Fischer and Fick, 1993; Strahan and Gerbasi, 1972) was included to account for this potential bias. A principal component-based factor analysis of this measure using pairwise deletion and constrained to a single-factor solution yielded an initial eigenvalue of 2.50, 25.02% of explained variance. Four items with loadings greater than 0.60 including “I like to gossip at times,” “There have been occasions when I took advantage if someone,” “I sometimes try to get even, rather than forgive and forget,” and “At times I have really insisted on having things my own way” were retained. A second factor analysis with the same criteria had an initial eigenvalue of 2.04, 51.09% of explained variance, and loadings greater than 0.680. This four-item measure of social desirability had acceptable internal consistency reliability (α = 0.68).

### Analysis

Using AMOS, confirmatory factor analysis (CFA) was specified to assess the multiple factor and multi-item measures, and a single factor test evaluated the potential for common method bias. Correlation analysis using SPSS was specified to assess the bivariate relationships among the variables (available upon request). Finally, mediation analyses with SEM and the PROCESS macro were utilized to test the hypothesized relationships among perceived stress, nonreactive mindfulness, and the EDM process.

## Results

The CFA yielded mostly acceptable fit statistics (CMIN = 342.43, DF = 183, *p* < 0.001, CMIN/DF = 1.87, NFI = 0.81, IFI = 0.90, CFI = 0.90, RMSEA = 0.06^1^), the observed items and composite scores positively related to the latent constructs (*p* < 0.001), all standardized regression weights were above 0.50, and all intercepts (*p* < 0.001) and error variances were significant (*p* < 0.05). The covariances/correlations were perceived stress↔individual mindfulness = −0.17 (*p* < 0.001)/−0.44, mindfulness↔EDM process = 0.09 (*p* < 0.01)/0.22, perceived stress↔EDM process = −0.05 (*p* = 0.355)/−0.07, perceived stress↔social desirability −0.23 (*p* < 0.001)/−0.37, mindfulness↔social desirability = 0.11 (*p* < 0.01)/0.29, and EDM process↔social desirability = 0.13 (*p* < 0.05)/0.20. Using standardized regression weights, composite reliability and average variance extracted scores for perceived stress (0.86/0.42), nonreactive mindfulness (0.71/0.34), EDM process (0.85/0.65), and social desirability (0.68/0.35) indicated sound reliability, but mixed variance captured. However, the squared correlations between for the variables were all below the variance extracted scores, showing adequate discriminant validity. The model for the single factor test yielded poor fit statistics (CMIN = 887.08, DF = 189, *p* < 0.001, CMIN/DF = 4.69, NFI = 0.51, IFI = 0.57, CFI = 0.56, RMSEA = 0.12), thus indicating that common method bias was likely not present (Podsakoff et al., 2024).

The correlation analysis is summarized in Table 1. The mean scores for perceived stress (*M* = 1.76), nonreactive mindfulness (*M* = 3.04), EDM process (*M* = 4.39), and social desirability (*M* = 3.99) indicated that (on average) individuals experienced modest levels of stress and exhibited slightly elevated nonreactive mindfulness, EDM process, and social desirability. As expected, perceived stress was negatively related to nonreactive mindfulness (*p* < 0.001). It was also significantly related to social desirability (*p* < 0.001). Nonreactive mindfulness was positively related to EDM process (*p* < 0.01), as expected, and it was also related to social desirability (*p* < 0.001). EDM process was also positively related to social desirability (*p* < 0.05).

**TABLE 1:** Variable descriptive statistics and correlations.

Variable	*M*	*SD*	*N*	1	2	3	4
1. Perceived stress	1.76	0.66	252	–	–	–	–
2. Nonreactive mindfulness	3.04	0.62	253	−0.39***			
3. Ethical decision-making process	4.39	0.84	210	−0.10	0.20**		
4. Social desirability	3.99	1.12	261	−0.31***	0.21***	0.15*	

***p < 0.001, **p < 0.01, *p < 0.05.

**FIGURE 1:**
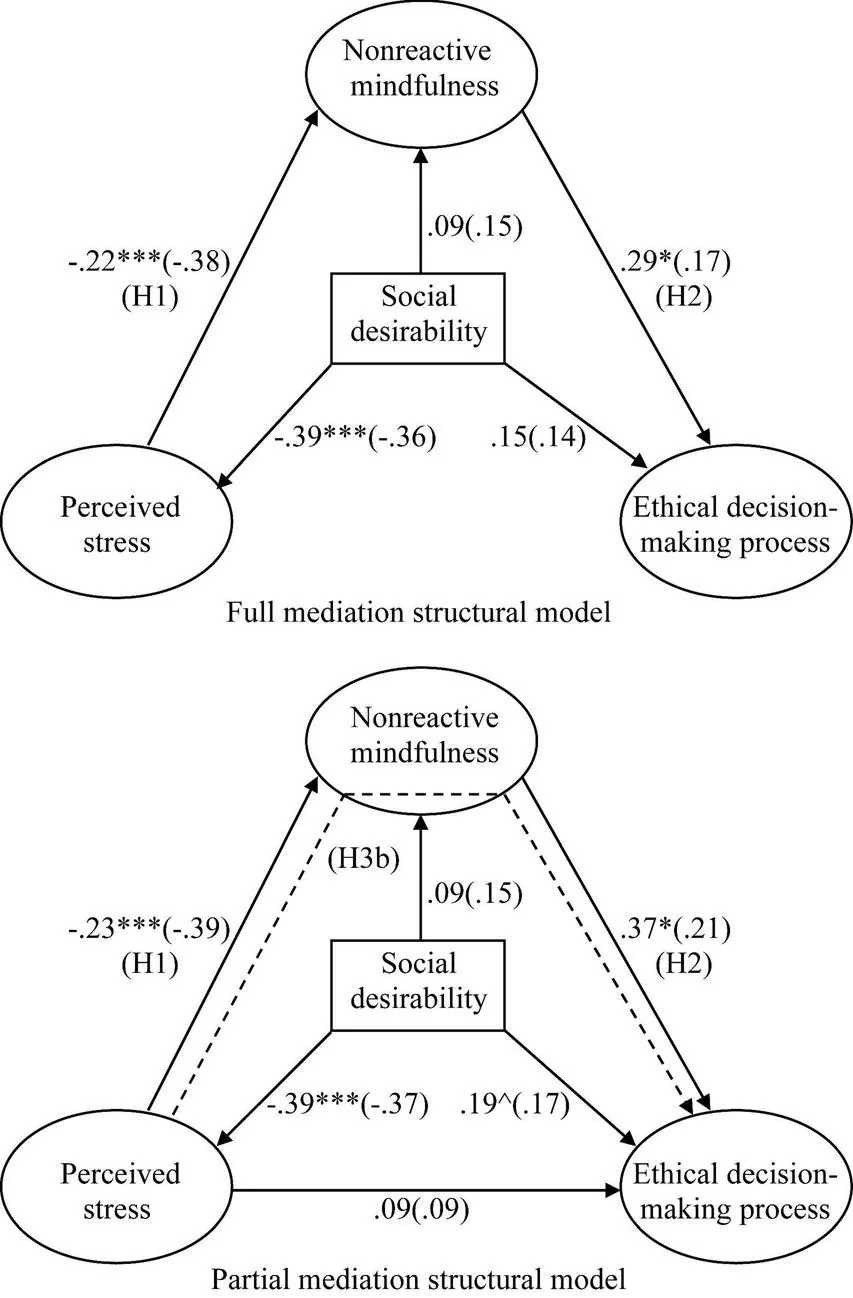
Mediation analysis; ***p < 0.001, *p < 0.05, ^*p* < 0.10; *N* = 261; H = hypothesis; dotted line denotes mediation; standardized parameter estimates presented in parentheses.

Results for the mediation analysis using SEM are presented in Figure 1. The full mediation structural model produced predominantly acceptable fit statistics (CMIN = 343.52, DF = 184, *p* < 0.001, CMIN/DF = 1.87, NFI = 0.81, IFI = 0.90, CFI = 0.90, RMSEA = 0.06), the regression weights associated with observed composite scores/scale items were positive and significant (*p* < 0.001); the associated standardized regression weights were all above 0.51, and parameter estimates associated with the intercepts (*p* < 0.001) and error variances were positive and significant (*p* < 0.05). Perceived stress was negatively related to nonreactive mindfulness (*p* < 0.001), and nonreactive mindfulness was positively related to the EDM process (*p* < 0.05), which supported Hypotheses 1 and 2. Social desirability was negatively related to stress (*p* < 0.001).

A partial mediation model was also evaluated. The partial mediation structural model also produced acceptable fit statistics (CMIN = 342.43, DF = 183, *p* < 0.001, CMIN/DF = 1.87, NFI = 0.81, IFI = 0.90, CFI = 0.90, RMSEA = 0.06). All the parameter estimates for the observed composite scores/scale items were acceptable and similar to those reported for the full structural model. Perceived stress was negatively related to nonreactive mindfulness (*p* < 0.001), and nonreactive mindfulness was positively related to the EDM process (*p* < 0.05), supporting H1 and H2. But the additional parameter between stress and the EDM process was insignificant and did not cause a significant reduction in the model chi-square (χ^2^ difference = 1.09, df = 1, *p* = 0.296). Consequently, statistical support for H3a was not secured, suggesting mediation in support of H3b.

While SEM provides parameter estimates and assessments of overall model fit, it does not provide a formal test of mediation. Therefore, mediation (H3b) was evaluated using the PROCESS modeling/bootstrapping approach (see Table 2). Perceived stress was negatively related to nonreactive mindfulness (*p* < 0.001), consistent with the results reported in the SEM analysis that supported H1. Nonreactive mindfulness was also positively related to the EDM process (*p* < 0.10), also supporting H2. While this relationship was marginally significant, a one-tailed test could be employed given the direction of the relationship was specified, suggesting that the significance was stronger. While the total and direct effects of perceived stress on the EDM process were insignificant, stress was negatively related to the EDM process by functioning through nonreactive mindfulness (p < 0.10), which provided statistical support for H3b. Once again, considering this was a two-tailed test, this marginally significant relationship was indeed stronger.

**TABLE 2:** Mediation analysis (perceived stress→nonreactive mindfulness→ethical decision-making process).

Outcome								
Model (independent variables)			Coefficient	se	*t*	*p*	LLCI	ULCI
Nonreactive mindfulness								
Constant			3.40	0.21	15.90	0.000	2.98	3.82
Perceived stress	(H1)		−0.30	0.06	−4.95	0.000	−0.42	−0.18
Social desirability			0.05	0.04	1.48	0.141	−0.02	0.13
	*R =* 0.39		MSE = 0.29		df1 = 2		*p* = 0.000	
	*R*^2^ = 0.15		*F* = 17.88		df2 = 196			
Ethical decision-making process								
Constant			3.57	0.48	7.41	0.000	2.62	4.52
Perceived stress			−0.03	0.10	−0.30	0.767	−0.22	0.16
Nonreactive mindfulness	(H2)		0.20	0.11	1.87	0.062	−0.01	0.41
Social desirability	*R =* 0.20		0.07	0.06	1.19	0.234	−0.04	0.17
			MSE = 0.65		df1 = 3		*p =* 0.052	
	*R*^2^ = 0.04		*F* = 2.61		df2 = 195			
Total effect model								
Ethical decision-making process								
Constant			4.24	0.32	13.26	0.000	3.61	4.88
Perceived stress			−0.09	0.09	−0.97	0.333	−0.27	0.09
Social desirability			0.08	0.06	1.39	0.166	−0.03	0.19
	*R =* 0.15		MSE = 0.65		df1 = 2		*p =* 0.120	
	*R*^2^ = 0.02		*F* = 2.14		df2 = 196			
Total effect of perceived stress on the ethical decision-making process			Effect	se	*t*	*p*	LLCI	ULCI
Direct effect of perceived stress on the ethical decision-making process			−0.09	0.09	−0.97	0.333	−0.27	0.09
			Effect	se	*t*	*p*	LLCI	ULCI
			−0.03	0.10	−0.30	0.767	−0.22	0.16
							Boot	Boot
Indirect effect of perceived stress on the ethical decision-making process	(H3b)		Nonreactive mindfulness		Effect	Boot SE	LLCI	ULCI
					−0.06	0.04	−0.14	0.003
Normal theory test for indirect effect			Effect	se	*Z*	*p*		
			−0.06	0.04	−1.72	0.085		

*N* = 199 (listwise); H = hypothesis.

Overall, the results support a fully mediated model in which perceived stress influences the EDM process indirectly through nonreactive mindfulness. These findings suggest that stress does not impair the EDM process directly but rather operates by reducing individuals’ capacity for nonreactive responses.

## Discussion

Previous studies of mindfulness and EDM have evaluated the relationship between mindfulness, overall, and specific stages of the EDM process. In contrast, we examined how a specific facet of mindfulness, nonreactive mindfulness, is related to the EDM process overall. As Selart and Johansen (2011) noted, “it is interesting to look at the whole EDM process, without making the distinction between recognizing ethical dilemmas and acting ethical” (p. 140).

H1 posited a negative relationship between perceived stress and nonreactive mindfulness: as stress rises, nonreactive mindfulness will decrease. Results from the analyses performed confirmed that, in this sample, increasing levels of chronic stress were associated with decreasing levels of nonreactive mindfulness. This result is consistent with theoretical perspectives (e.g., conservation of resources theory; Hobfoll, 1989) predicting that increased stress will consume cognitive and emotional processing bandwidth, leaving less available for other psychological processes – in this case, nonreactive mindfulness.

H2 stated that nonreactive mindfulness would be positively related to EDM, and our analysis confirmed the expected effect. This result is generally consistent with previous studies (Ming et al., 2025; Ruedy and Schweitzer, 2010; Small and Lew, 2019) determining that mindfulness had a positive effect on one or more stages of EDM. Our investigation of the influence of the specific nonreactive facets of mindfulness is a more nuanced approach in the study of EDM and adds to the literature by identifying a specific component of mindfulness and further developing theory by explaining why it is related to EDM. The results of the nonreactive mindfulness–EDM process relationship are generally consistent with the benefits of workplace mindfulness (e.g., Good et al., 2016), as nonreactive mindfulness has a positive relationship with the desired outcome, in this case, improved EDM. One implication of these results is that a skill-based path (e.g., mindfulness training, specifically oriented toward nonreactive mindfulness) could improve students’ and working professionals’ EDM capabilities, in addition to instruction in ethics. We reiterate the assertions of Giacalone and Promislo (2019), as highlighted in Jeong et al. (2020), p. 303), specifically that “Business educators have the responsibility to inform and guide students to make better decisions” and suggest that this responsibility also applies to employers in the workplace.

We hypothesized that stress would either operate directly (H3a) and indirectly through nonreactive mindfulness (partial mediation), or that its effects upon the EDM process would be fully mediated (H3b) through nonreactive mindfulness. The full mediation model fits the data better than the partial mediation model and this result is important to consider. First, it confirms that adverse effects of stress appear to covary with the EDM process by reducing the individual’s nonreactive mindfulness capacity. Since participants were engaged in a stressful life stage, their cognitive and affective resources may be consumed with reduced capacity for EDM. Full mediation suggests that while stress may not typically directly affect EDM, it can affect it indirectly through nonreactive mindfulness.

Full mediation is consistent with Selart and Johansen (2011), where they found very limited evidence of a direct effect of stress on EDM. It is also conceptually similar to Stenmark and Kreitler (2019) who, contrary to expectations, did not find evidence of an effect of externally imposed pressure on EDM in a lab setting. Those results stand in contrast to Boals and Banks (2012), who found that perceived stress had a significant effect upon “everyday cognition,” after controlling for other factors. Noting the typical effect of stress upon cognition, Boals and Banks (2012) stated, “There has been a plethora of research examining the relationship between stress and cognition. Much of this research has focused on laboratory-based tasks as measures of cognitive ability” (p. 1335). This survey-based study measured chronic stress, rather than an induced state of stress, which is typical of laboratory studies (e.g., Boals and Banks, 2012; Stenmark and Kreitler, 2019). It is conceivable that the effects of chronic stress on the EDM process are manifested differently than an induced state of acute stress.

Interestingly, Selart and Johansen (2011) found stress had little influence on recognition of moral issues, and a relatively small overall effect of stress on the EDM process when pooling data and effects. However, their examination of 10 different situations revealed stress was sometimes positively related to unethical behaviors in some situations, while negatively related to EDM in others. This observation provides support for a nuanced view in which situational characteristics may mediate or moderate the effects of stress upon EDM. We pooled data from three ethical scenarios, and it is plausible that, like Selart and Johansen (2011), the effect of specific stressors was obscured within pooled data.

### Limitations

It is important to note that this study made no attempt to manipulate any aspect of mindfulness. Given the plethora of existing research on manipulation of state mindfulness, as an intervention, it is conceivable that a mindfulness intervention may be able to reduce or mitigate the adverse (direct and/or indirect) effects of stress on mindfulness and EDM. Future research studies may want to consider this.

We also note that prior research suggests that as stress increases from relatively low to more moderate levels, individuals are typically able to effectively manage stress and may perform better due to heightened awareness and focus. However, higher levels of stress are likely (in the absence of significant support and/or compensating mechanisms) to result in significant reductions in otherwise routine mental capacity, emotional control, and are likely to adversely impact individuals physiologically. Under these circumstances, the expected result is a decrease in the level of self-reported nonreactive mindfulness. The transition to higher levels of stress may also result in a transition from cognitive to emotional/intuitive processing and thus, lower reported nonreactive mindfulness. From the data collected, it is not possible to determine whether the levels of stress reported in this study follow a non-linear effect pattern with respect to either mindfulness practice or the EDM process.

Stress is widespread among young adults, especially those in the workplace and in university students. Individuals often make ethical decisions in a chronic or acutely stressed state (Singer et al., 2017). Even encountering ethical scenarios induces stress (Kälvemark et al., 2004). However, the ethical scenarios in the current study may not be particularly stress-inducing, thereby lacking ecological and external validity and not generating direct effects. The current study involved chronic stress in university students, most of whom are also working. High levels of acute stress might lead to increased cognitive and emotional challenges in EDM. Importantly, both high chronic and acute stress negatively influence complex and flexible reasoning (Sandi, 2013).

A related limitation concerns the nature of the stress examined. Although students, especially those individuals working full-time, often experience chronic stress, they evaluated hypothetical ethical scenarios rather than making decisions with real organizational consequences. As a result, participants may not have experienced the same intensity or type of stress, or the level of emotional involvement, that typically accompanies workplace ethical dilemmas. Accordingly, these findings should be interpreted with caution. Future research should extend this work to organizational settings, where ethical decisions carry real consequences and stress is more directly tied to those outcomes.

Similarly, levels and variation of nonreactive mindfulness were limited. Furthermore, only trait and not state mindfulness was evaluated in the current study. The development of nonreactive mindfulness enables enhanced and improved capacity for improved ethical problem-solving and decision-making, even under stress. The present study did not seek to develop or induce nonreactive mindfulness, rather it assessed relative presence/absence in participants. Finally, we note that data is cross-sectional. Thus, we cannot make any certain conclusions about causality. Future research might induce state mindfulness and consider causality longitudinally.

### Practical implications and future research directions

One potentially interesting avenue of investigation would involve examination of the effects of short and long-term mindfulness intervention(s) on the EDM process for young adults who are students and/or working professionals. Anecdotally, the author(s) are aware of other university faculty who weave mindfulness training into their courses and have (anecdotally) reported positive results. Adapting and applying mindfulness interventions and investigating effects on students’ EDM process could yield valuable insights.

Future work might also study the EDM process in young adults, under high levels of chronic or acute stress. Stressed individuals may be more prone to moral disengagement, essentially rationalizing unethical behavior or distancing themselves from the ethical implications of their actions. Some research has found benefits of leaders’ mindfulness on employees (Liu et al., 2024), and this influence could extend to EDM in higher education as well as the professional workplace. Further, data should be collected at multiple time periods or through experimental procedures to provide more robust inferences of causality (Stone-Romero and Rosopa, 2008). Future investigation may reveal an improved understanding of the impact of stress and mindfulness on ethical behavior, particularly considering dual-process EDM approaches, to inform both researchers and business faculty and practitioners.

Although the present findings suggest that nonreactive mindfulness may serve as an important resource to enhance individual-level ethical awareness and self-regulation, ethical behavior is influenced at multiple levels. Consistent with Jones’s (1991) contingency model, EDM is shaped not only by individual factors but also by contextual factors such as organizational culture, incentive systems, and institutional constraints. Further, organizations and leaders are also responsible for ethical business decisions. Therefore, promoting individual mindfulness alone as a means of addressing organizational ethics would miss the broader issue of the multi-level context of unethical behaviors. Therefore, individual mindfulness-based approaches would ideally operate as complement rather than replacement for supports to strengthen ethical climates and governance structures in organizations and broader societal efforts.

## Data Availability

The raw data supporting the conclusions of this article will be made available by the authors, without undue reservation.

## Footnotes

1 CMIN, chi-square; DF, degrees of freedom; CMIN/DF, relative chi-square; NFI, normed fit index; IFI, incremental fit index; CFI, comparative fit index; RMSEA, root mean square of approximation.
